# Cardiac Arrest in the Post-anesthesia Care Unit (PACU): Postoperative Recurrence of Neuromuscular Block After Sugammadex Reversal

**DOI:** 10.7759/cureus.52681

**Published:** 2024-01-21

**Authors:** Vasyl Katerenchuk, Alexandre Calçada, Raquel Louzada, Daniela Rosinha, Ana C Batista, Andreia Capelão, Lisbete Cordeiro

**Affiliations:** 1 Anesthesiology, Centro Hospitalar de Setúbal E.P.E., Setúbal, PRT

**Keywords:** monitoring, recurarization, rocuronium, neuromuscular blockade, sugammadex, cardiac arrest

## Abstract

Sugammadex has transformed clinical practice by enabling the rapid reversal of rocuronium-induced neuromuscular block (NMB) at any depth. We present a case of cardiac arrest following postoperative recurarization despite the sugammadex-induced transient reversal of NMB. Despite its proven clinical reliability, this case highlights the often overlooked aspects that must be considered when using this drug. An 84-year-old male patient was scheduled for a laparoscopic partial gastrectomy for gastric cancer. At the end of the procedure, reversal of NMB was evidenced by an acceleromyographic train-of-four (TOF) ratio of ≥0.9 following sugammadex administration. In the post-anesthesia care unit (PACU), pulseless electrical activity was perceived, with a regression of TOF count to 1. After providing successful advanced cardiac life support, additional sugammadex administration led to uneventful extubation.

When the concentration of free rocuronium decreases in the central compartment following sugammadex administration, redistribution of rocuronium from the peripheral to the central and effect-site compartments may cause recurarization. Special care is required in cases involving obese and elderly patients as well as those with renal impairment or hypothermia. To provide effective and predictable reversal of NMB, proper use of sugammadex should be pursued, including adequate dosing and monitoring.

## Introduction

The introduction of sugammadex changed the clinical practice by allowing rapid reversal of rocuronium- and vecuronium-induced neuromuscular block (NMB) at any depth [1,2]. Unlike traditional acetylcholinesterase inhibitors, sugammadex, a modified gamma-cyclodextrin, functions as a selective relaxant binding agent, ensuring a faster reversal of NMB without the associated muscarinic side effects. Despite its clinical reliability, it is important to take precautions when using it, particularly regarding the dosage and concomitant NMB monitoring [3]. We present a case of cardiac arrest following postoperative recurarization despite the sugammadex-induced complete reversal of NMB, a rarely reported occurrence.

This case report was previously presented as a meeting abstract at ANESTHESIOLOGY 2022, New Orleans, LA.

## Case presentation

The patient was an 84-year-old male scheduled for a laparoscopic partial gastrectomy for gastric cancer. His medical history included arterial hypertension, chronic kidney disease grade 3b, paroxysmal atrial fibrillation, ischemic stroke one year prior (no sequelae), dyslipidemia, mild anemia, and overweight (weight: 77 kg; height: 166 cm; BMI: 28 kg/m²).

Surgery was performed under total intravenous anesthesia. Tracheal intubation was administering 100 mg of succinylcholine followed by a bolus of 40 mg of rocuronium. NMB was maintained with continuous rocuronium infusion at a rate of 0.3 to 0.7 mg·kg^-1^·h^-1^ targeting a deep NMB to enhance surgical conditions. The American Society of Anesthesiologists (ASA) standards for invasive blood pressure, Bispectral Index™, and quantitative NMB monitoring were assessed. Train-of-four (TOF) acceleromyography monitor (ToFscan®, Dräger Medical, Lübeck, Germany) was used, after the placement of stimulating electrodes over the ulnar nerve, measuring the evoked adductor pollicis muscle response throughout surgery with a five-minute interval. The radial arterial line and right internal jugular central venous line were placed uneventfully. Despite the need to convert to a laparotomy, the surgery was uneventful.

Rocuronium continuous infusion was stopped 60 minutes before the end of surgery after a total of 200 mg was administered. At the end of the procedure, which lasted five hours and 30 minutes, 400 mg (5.2 mg/kg) of sugammadex was administered to reverse a deep level of NMB (no responses to TOF stimulation). A TOF count of 4 and a TOF ratio >0.9 were evidenced repeatedly by the ToFscan monitor thereafter. Active warming by forced expired air, heating of crystalloid fluids, and measurement of core temperature with an oropharyngeal probe were maintained during surgery. However, inadvertent postoperative hypothermia was observed (33.4 °C).

Following uneventful extubation, the patient was transferred to the postanesthesia care unit (PACU) fully awake; he responded to verbal commands and was able to breathe spontaneously. Upon arrival at the PACU, the patient was hemodynamically stable, normoxemic, and without pain or nausea. The temperature measured by the infrared scanner was 34.5 °C and a forced warmed air blanket was applied. Twenty minutes after the PACU admission, and approximately half an hour after the reversal of NMB, pulseless electrical activity was observed. Immediate cardiopulmonary resuscitation was initiated, 1 mg of epinephrine was administered, and tracheal intubation was performed without the administration of neuromuscular blockers. The return of spontaneous circulation ensued within two minutes. The patient was kept under mechanical ventilation and sedation was started. Initial arterial blood gas assessment showed a slight respiratory acidosis (probably due to apnea), and there were no significant findings on EKG, chest radiograph, and blood tests (including troponin values).

At that point, an acceleromyography neuromuscular monitoring device at the adductor pollicis muscle (TOF-Watch®, Organon Teknika Corporation, NC) was applied and showed a TOF count of 1, in three different attempts; 200 mg of sugammadex (2.6 mg/kg) were administered and, about two minutes later, the patient showed spontaneous breathing efforts with TOF-Watch® readings showing a TOF count of 4 with a TOF ratio 0 .9. Following the cessation of sedation, the patient responded to verbal commands. The trachea was extubated with the patient fully awake. A brief neurological examination showed no acute impairment. The patient was subsequently transferred to the ICU, where he stayed for another 24 hours uneventfully.

## Discussion

Studies have defined recurarization (or recurrent NMB) as the decrease of the previous TOF ratio of ≥0.9 to <0.8 over three consecutive measurements [4,5]. In this report, certain aspects contributed to the diagnosis of recurarization after sugammadex administration: (a) repeated objectively measured TOF ratio >0.9 in the operating room before emergence; (b) the patient was capable of maintaining spontaneous ventilation and responded to verbal commands at extubation and on arrival to the PACU; (c) possible cardiac arrest due to respiratory failure; (d) a lack of any other clear etiology for cardiac arrest; (e) TOF count of 1 in the PACU; and (f) the positive response to additional sugammadex with TOF count improvement and successful extubation.

The recurrence of NMB could be attributed to the lack of a sufficient number of unbound sugammadex molecules to the total rocuronium present in the body (at the effect-site, central, and peripheral compartments) [6,7]. Eventually, the first dose of 5.2 mg/kg sugammadex was enough to initially encapsulate the rocuronium molecules present in the central (intravascular) and effect-site (neuromuscular junction) compartments, restoring neuromuscular function [5-9]. However, when the concentration of free rocuronium decreased in the central compartment, redistribution of rocuronium from the peripheral to the central and effect-site compartments probably occurred due to concentration gradients [6-8]. Hence, unbound rocuronium was available to move back into the neuromuscular junction, down a concentration gradient. In the absence of free sugammadex molecules, this free rocuronium may have caused a recurrence of NMB [5-7,9]. Therefore, probably the first dose of sugammadex was insufficient taking into account the total body amount of rocuronium accumulated during continuous infusion. 

Case reports and randomized trials have associated smaller doses of sugammadex with decreases in TOF count after the reversal of NMB [4,5,8,9]. In one randomized trial that assessed the appropriate dosing of sugammadex to reverse deep rocuronium-induced NMB [post-tetanic count (PTC) of 1 to 5], recurarization occurred in the middle-dose arm (2 mg/kg), whereas most of the patients in the low-dose arm (1 mg/kg) needed an additional dose of sugammadex but did not experience a reversal followed by recurarization [4]. This supports the hypothesis that recurarization occurs in a limited range of intermediate suboptimal doses of sugammadex, also endorsed by one simulation model [8]. This further enhances the importance of administering an adequate quantity of sugammadex to encapsulate all the molecules of rocuronium present in the central and peripheral compartments, thereby avoiding the occurrence of unbound free rocuronium [7].

Although we cannot directly measure the quantity of rocuronium present in the body, we can estimate this value through neuromuscular function monitoring to determine the adequate dosage of sugammadex [7,9]. Excessive doses of sugammadex may prevent residual NMB or recurarization at the expense of increased costs and hampering the easy use of rocuronium in the immediate postoperative period [7]. Fortunately, unbinding of sugammadex-rocuronium complexes is unlikely, since one molecule of sugammadex binds with high affinity to one molecule of rocuronium [8,10].

Recurarization after reversal of NMB with sugammadex has been previously reported in a few trials. Reports involving obese patients have recommended underdosing by considering total body weight [4,10]. Higher BMI has been associated with slower recovery from muscle paralysis after low-dose sugammadex administration [11]. Evidence has consistently suggested that sugammadex dose administration to obese patients should be based on actual body weight [3,4,7,12]. Patients’ advanced age could be an additional risk for recurarization, as one article has shown that small doses of sugammadex were associated with an increased risk of residual paralysis and recurarization in the elderly (≥70 years old) [11].

Another report has described recurarization after repeated administration of sugammadex following a prolonged rocuronium infusion to prevent shivering during induced therapeutic mild hypothermia [6]. Hypothermia was also associated with prolonged recovery from NMB [7]. Several risk factors might have had an impact on our case, as depicted in Table 1, such as the patient’s age and weight, hypothermia, and moderately impaired renal function, which has also been previously associated with recurarization [11]. Moreover, continuous rocuronium infusion throughout a prolonged surgical procedure eventually leads to the accumulation of an excessive dose of rocuronium.

**Table 1:** Risk factors for recurarization in our case report NMB: neuromuscular block; PTC: post-tetanic count

Risk factors
Suboptimal intraoperative NMB monitoring (with no PTC obtained)
Potentially incorrect sugammadex dosing if the PTC is 0
Hypothermia
Overweight
Impaired renal function

This case report has a few limitations. After the procedure, following the lack of response to TOF stimulation, PTC was not monitored before the reversal of NMB. Although the rocuronium infusion was stopped 60 minutes before sugammadex administration, measurement of PTC would have been relevant in the setting of a deep NMB to exclude the possibility of a complete block (PTC of 0). Also, due to equipment availability, two different neuromuscular transmission monitors (one in the operating room and one in the PACU) were employed, although both provided quantitative acceleromyography measurements.

Recurrence of NMB following sugammadex administration using the dose labeled for the depth of NMB has an estimated incidence of 0.2% [3]. To ensure rapid, effective, and predictable reversal of moderate and deep NMB, the proper use of sugammadex should be pursued, including adequate dosing and NMB monitoring. Special care must be taken when dealing with certain populations, such as obese and elderly patients and those with renal impairment.

## Conclusions

Quantitative NMB monitoring is recommended throughout the surgical course (including PTC monitoring when deep NMB is desired) to avoid underdosing of sugammadex and, consequently, possible complications related to recurarization and residual blockade. Moreover, we should always consider the risk factors for recurrence and/or residual paralysis. Finally, recurarization should be considered in the differential diagnosis of cardiac arrest or ventilatory distress in patients who received sugammadex despite evidence of NMB reversal.

## References

[1] Fuchs-Buder T, Romero CS, Lewald H (2023). Peri-operative management of neuromuscular blockade: a guideline from the European Society of Anaesthesiology and Intensive Care. Eur J Anaesthesiol.

[2] Thilen SR, Weigel WA, Todd MM (2023). 2023 American Society of Anesthesiologists Practice Guidelines for monitoring and antagonism of neuromuscular blockade: a report by the American Society of Anesthesiologists Task Force on Neuromuscular Blockade. Anesthesiology.

[3] (2024). Bridion. European Medicines Agency. https://www.ema.europa.eu/en/medicines/human/EPAR/bridion.

[4] Loupec T, Frasca D, Rousseau N, Faure JP, Mimoz O, Debaene B (2016). Appropriate dosing of sugammadex to reverse deep rocuronium-induced neuromuscular blockade in morbidly obese patients. Anaesthesia.

[5] Duvaldestin P, Kuizenga K, Saldien V (2010). A randomized, dose-response study of sugammadex given for the reversal of deep rocuronium- or vecuronium-induced neuromuscular blockade under sevoflurane anesthesia. Anesth Analg.

[6] Murata T, Kubodera T, Ohbayashi M, Murase K, Adachi YU, Matsuda N (2013). Recurarization after sugammadex following a prolonged rocuronium infusion for induced hypothermia. Can J Anaesth.

[7] Iwasaki H, Renew JR, Kunisawa T, Brull SJ (2017). Preparing for the unexpected: special considerations and complications after sugammadex administration. BMC Anesthesiol.

[8] Eleveld DJ, Kuizenga K, Proost JH, Wierda JM (2007). A temporary decrease in twitch response during reversal of rocuronium-induced muscle relaxation with a small dose of sugammadex. Anesth Analg.

[9] Fuchs-Buder T (2010). Less is not always more: sugammadex and the risk of under-dosing. Eur J Anaesthesiol.

[10] Le Corre F, Nejmeddine S, Fatahine C, Tayar C, Marty J, Plaud B (2011). Recurarization after sugammadex reversal in an obese patient. Can J Anaesth.

[11] Muramatsu T, Isono S, Ishikawa T (2018). Differences of recovery from rocuronium-induced deep paralysis in response to small doses of sugammadex between elderly and nonelderly patients. Anesthesiology.

[12] Horrow JC, Li W, Blobner M, Lombard J, Speek M, DeAngelis M, Herring WJ (2021). Actual versus ideal body weight dosing of sugammadex in morbidly obese patients offers faster reversal of rocuronium- or vecuronium-induced deep or moderate neuromuscular block: a randomized clinical trial. BMC Anesthesiol.

